# Tolerance and response of two honeybee species *Apis cerana* and *Apis mellifera* to high temperature and relative humidity

**DOI:** 10.1371/journal.pone.0217921

**Published:** 2019-06-06

**Authors:** Xinyu Li, Weihua Ma, Jinshan Shen, Denglong Long, Yujia Feng, Wenting Su, Kai Xu, Yali Du, Yusuo Jiang

**Affiliations:** 1 College of Animal Science and Technology, Shanxi Agricultural University, Taigu, Shanxi, China; 2 Horticulture Institute, Shanxi Academy of Agricultural Sciences, Taiyuan, Shanxi, China; 3 Apiculture Science Institute of Jilin province, Jilin, Jilin, China; University of North Carolina at Greensboro, UNITED STATES

## Abstract

The ambient temperature and relative humidity affect the metabolic and physiological responses of bees, thus affecting their life activities. However, the physiological changes in bee due to high temperature and high humidity remain poorly understood. In this study, we explored the effects of higher temperature and humidity on the epiphysiology of bees by evaluating the survival, tolerance and body water loss in two bee species (*Apis cerana* and *Apis mellifera*). We also evaluated the changes in the activity of antioxidant and detoxification enzymes in their body. We observed that under higher temperature and humidity conditions, the survival rate of *A*. *mellifera* was higher than that of *A*. *cerana*. On the other hand, a comparison of water loss between the two species revealed that *A*. *mellifera* lost more water. However, under extremely high temperature conditions, *A*. *cerana* was more tolerant than *A*. *mellifera*. Moreover, under higher temperature and humidity conditions, the activity of antioxidant and detoxification enzymes in bees was significantly increased. Overall, these results suggest that high temperatures can adversely affect bees. They not only affect the survival and water loss, but also stimulate oxidative stress in bees. However, unlike our previous understanding, high humidity can also adversely affect bees, although its effects are lower than that of temperature.

## Introduction

Honey bees are poikilothermal animal. The ability of bees to maintain and regulate body temperature is poor. The ambient temperature affects the metabolic and physiological responses of bees, and thus, affecting in their life activities [1,2,3] such as growth, development, reproduction and survival [4,5,6]. Furthermore, it decreases the hatching rate, deforms the wings and decreases the learning ability of bees [7], as well as increases their susceptibility to infection [8]. In addition, high ambient temperatures significantly inhibits the foraging activity of bees [9,10].

Relative humidity (RH) has a significant effect on larvae during incubation [11]. It has been reported that approximately 75% RH is the most suitable for larval hatching [12]. When bees are outside the colony, the change in humidity has no direct effect on the feeding behavior of bees [13]. Worker bees stably maintain the humidity of a colony by various methods, including evaporation of nectar water and water collection of water [11]. Therefore, maintaining stable temperature and RH is important for the activity of bees.

Honeybees, known for their importance as pollinators in natural habitats, have long-term co-evolution with plants, and their biological characteristics are perfectly harmonious in color, aroma, and structure [14]. In recent years, the introduction of bees into greenhouses for pollination has not only reduced the cost of artificially assisted pollination, but also significantly increased fruit set rate and yield [15]. However, the small space in the greenhouse and the higher temperature and humidity have impact on the foraging behavior of bees, as well as their reproduction [16].

Temperature stress can induce oxidative stress in insects, and their antioxidase play a major role against oxidative stress [17]. Under high temperature stress, insects undergo anti-oxidation reactions, remove excess oxygen free radicals from the body, and maintain normal physiological activities [18–19]. Insects have a range of antioxidant enzymes, mainly composed of superoxide dismutase (SOD), catalase (CAT), and peroxidase (POD). In addition, glutathione peroxidase (GPX), and glutathione reductase (GSR) can also remove oxygen peroxide [20]. Detoxification enzymes play critical crucial role in the biochemical defense system of insects. Insects mainly product clofenotane dehydrochlorinase (DDTase), glutathione S-transferase (GST), carboxylesterase (CES), cytochromeP450 (CYP450P), and acetyl cholinesterase (AChE) for detoxification [20,21]. The antioxidant enzyme system can effectively eliminate active oxygen from the body, and the detoxification enzyme system can help resist the damage caused by metabolic poisons and harmful substances. These systems are in equilibrium under normal conditions, however, environmental stresses such as high temperatures can disrupt this balance, causing cell damage or even death [22].

*A*. *cerana* and *A*. *mellifera* are the two most commonly cultured bee species. They are also widely used for pollination in agricultural facilities [23]. Currently, studies on the effect of environmental conditions of a facility on bees are limited. Moreover, the effects of higher temperature and humidity on the physiology of bees and the differences in the effects according to bee species are less reported. In the preset study, *A*. *cerana* and *A*. *mellifera* were used as research objects. We explored the changes in the physiological activities of honeybees under high temperature and humidity conditions, and compared their adaptability.

## Materials and methods

### Colonies and bees

The study was conducted at the Shanxi Agricultural University (Taigu, Shanxi, China) from June to August 2018. We selected three healthy colonies of *A*. *cerana* and *A*. *mellifera* for the study. For the epiphysiological experiment, we chose foragers that returned to the hive. For the enzyme activity experiment, we selected 1–2 capped brood combs from the colony and placed it in an incubator at 34°C and 75%RH. After emergence from the cell, the bee were marked on the back with non-toxic, odorless paint, returned to the original colony, and then sampled at the age of 20 days.

### Experiment 1 epiphysiology

#### Survival rate

For the experiment, we used a 16cm × 7cm × 7cm wooden box with one side covered by a transparent plastic plate. Thirty workers per each cage per species were used per treatment. 5 mL of water is provided to the bees in each box, 1mL 30% syrup. We chose the temperatures and humidity levels based on the temperature and humidity data of the greenhouse. Temperatures of 35°C, 40°C, and 45°C at constant RH, and RH levels of 50%, 60%, and 70% at constant temperature were tested. The following experiments were conducted under controlled conditions of temperatures and RH in incubators (Le Dian, Ningbo, China). The number of dead workers was counted every hour under different treatment conditions. Worker survival rate per each cage was calculated as the total number of hours at which all bees had died. Subsequently, the mean survival rate per treatment was calculated by dividing the total number of hours at which all the bees died in the three cages by three.

#### Temperature tolerance

We used the method of Atmowidjojo [24] to evaluate the tolerance of the two species, with appropriate modifications. A total of 150 bees per species were used in this experiment (50 bees per cage and three cages per species). The incubator was maintained at constant humidity (50%) and the temperature was programmed to start at 30°C and increase to 70°C during a 60-min period. After the starts of the experiment, the number of bees that were intolerant and dead for every 1°C increase in temperature was recorded. The dorsal turning reflex was used to assess temperature tolerance. The bees that were unable to turn toward their right immediately were classified as intolerant to a given temperature. The temperature at which the bees exhibited intolerance was recorded.

#### Loss of body water

For the experiment, we used a 10mL centrifuge tube with numerous small holes and one bee was placed in each tube (30 bees per species per treatment). The bees were weighed using a balance (Shunyu Hengping, China) to the nearest 0.1 mg (W1). The temperature and humidity selected for this experiment were consistent with those of the survival rate experiment. The test bees were maintained at a certain temperature and humidity for 2 hours (the choice of temperature and humidity is consistent with the survival rate experiment) and then reweighed (W2). The water loss was calculated as (W1)–(W2). To determine the water loss per bee, each weight was divided by 10 and the results are expressed as mg h –1. The rate of water loss was calculated as (W1)–(W2) / (W1).

### Experiment 2 antioxidant enzyme activities

For this experiment, we used 20-day-old bees, which were placed in separate 10-mL centrifuge tubes and treated for 2 h at different temperatures and humidity. We set up four different treatments: control treatment (CK: 25°C 30% RH), high temperature treatment (T: 45°C 30% RH), high humidity treatment (RH: 25°C 80% RH), and high temperature and high humidity treatment (TH: 45°C 80% RH).

Ten samples were taken from the whole tissue and ground with liquid nitrogen. To this mixture, a certain amount of PBS (pH = 7.4) was added and mixed by vortexing. The mixture was then centrifuged at 2000–3000 rpm for approximately 20 min and the supernatant was collected carefully. (1) Standard sample loading: standard and sample wells were set (standard wells plus 50μL of different concentrations of standard); (2) Loading: The sample wells to be tested and the blank wells were set separately (the blank control wells were not added with the sample and the enzyme standard reagent, the other steps were the same). Approximately 40 μl of the sample dilution was added to each sample well, and the test was performed on an enzyme-labeled plate, followed by the addition of 10 μl of the sample to be tested (the final dilution of the sample was 5 times). (3) Addition of enzyme: To each well, 100 μl of enzyme labeling reagent was added, except for blank wells. (4) Incubation: After sealing with a sealing membrane, the plates were incubated at 37° C for 60 min. (5) Dosing: The 20-fold concentrated washing solution was diluted 20 times with distilled water and used. (6) Cleaning: The sealing film was carefully removed, the liquid was discarded, and the wells were allowed to dry; thereafter, each well was filled with the cleaning solution, allowed to stand for 30 seconds, then discarded; this step was repeated 5 times. (7) Color development: First, 50 μl of developer A was added to each well, followed by 50 μl of the developer B; thereafter, the mixture was gently shaken and mixed, and colored at 37° C for 15 min. (8) Termination: A 50 μl sample of stop solution was added to each well to stop the reaction. (9) Measurement: Zeroing was performed with blank holes, and the absorbance (OD) of each well was measured sequentially at the corresponding wavelength.

The activity of five enzymes, namely, SOD, CAT, POD, GPX, and GSR was tested using their respective assay kits (Jianglai Biological, China).

### Experiment 3 detoxification enzyme activities

The same method was followed as described for antioxidant enzyme activities. The activity of five enzymes, DDTase, GST, CES, CYP450, and AChE, was tested using their respective assay kits (Jianglai Biological, China).

### Statistical analyses

The study was designed using the principle of complete randomization. The data were statistically analyzed by the analysis of variance (ANOVA) and the means were compared by the least significant difference test (LSD p<0.05).

## Results

### Epiphysiology

#### Survival rate

The survival of the two species under different temperature and humidity conditions showed that the survival time of the *A*. *mellifera* was slightly higher than that of the *A*. *cerana*. A significant difference (P < 0.05) was found between the survival rate of *A*. *cerana* and *A*. *mellifera* at 35°C and 60% RH, whereas, no significant differences were found among the other treatments (Table 1).

**Table 1:** Mean survival (hours) of two honeybee species under different temperature and relative humidity treatments.

RH(%)		Survival mean (hours) ± SE		
	T(°C)	*Apis cerana*	*Apis mellifera*	LSD_0.05_
50	35	11.54±2.45	11.21±0.68	0.94
	40	5.54±0.23	5.12±0.07	0.77
	45	2.02±0.17	2.91±0.13	0.66
60	35	4.14±0.25	5.78±0.36	0.002
	40	3.69±0.17	4.26±0.56	0.23
	45	2.34±0.06	3.22±0.12	0.07
70	35	3.56±0.36	4.58±0.23	0.27
	40	3.48±0.47	4.05±0.40	0.22
	45	1.67±0.87	2.16±0.75	0.08

**Fig 1:**
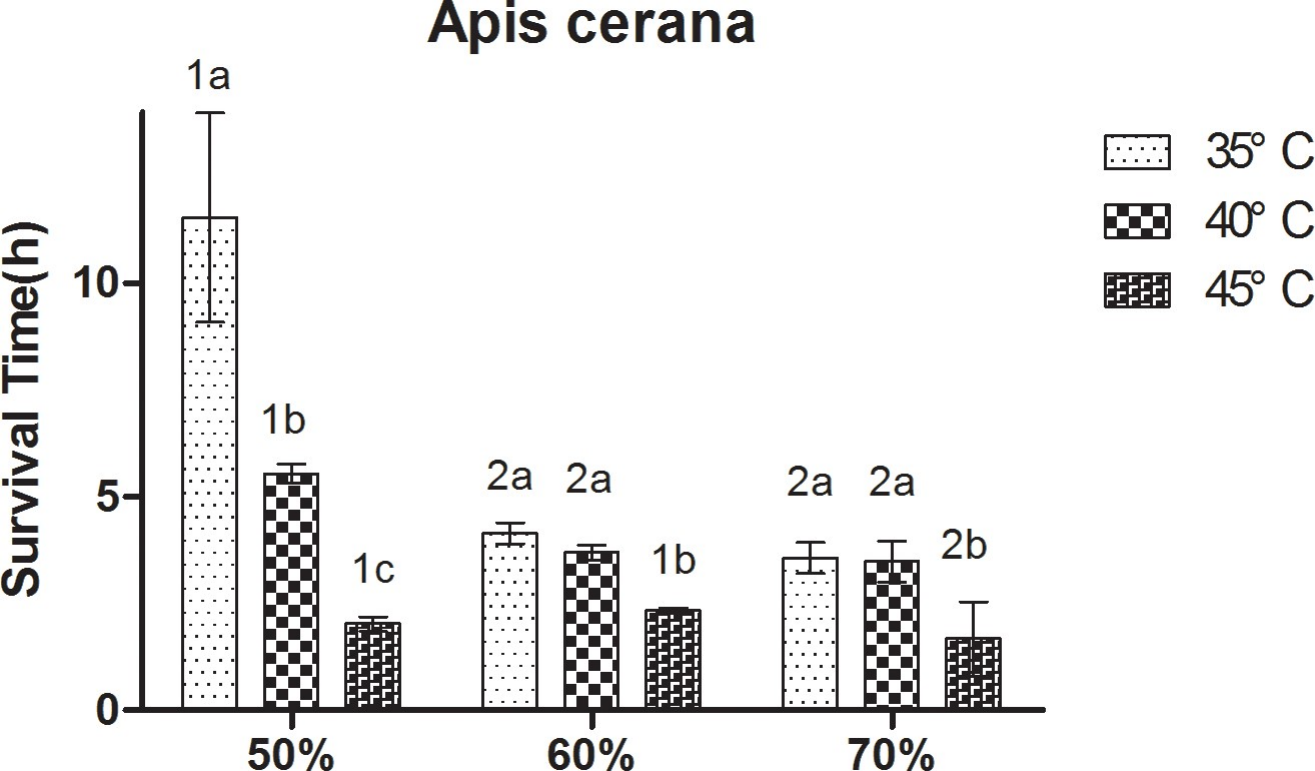
Mean ± SE survival (h) of *Apis cerana* under different temperature and relative humidity treatments. The lowercase letters indicate significant differences between *A*. *cerana* under different temperatures with constant humidity and the numbers indicate significant differences under different humidity levels with constant temperatures.

At constant humidity, the survival rate of bees decreased with increasing temperature. A significant (P < 0.05) and strong negative correlation was observed between survival rate and temperature (Fig 1). At 50% RH, *A*. *cerana* showed a relatively long survival rate at 35°C, followed by that at 40°C, and the survival time of *A*. *cerana* was the shortest at 45°C. Significant differences between the three treatments were observed (P < 0.05). At 60% RH and 70% RH, the survival rate of *A*. *cerana* was significantly lower at 45°C than at 35°C and 40°C. At 60% RH, *A*. *mellifera* showed a relatively long survival rate at 35°C, followed by that at 40°C, and the survival time of *A*. *mellifera* was the shortest at 45°C. Significant differences between the three treatment were observed (P < 0.05). At 50% RH, the survival rate of *A*. *mellifera* was significantly lower at 40°C and 45°C than at 35°C. At 70% RH, the survival rate of *A*. *mellifera* was significantly lower at 45°C than at 35°C and 40°C.

**Fig 2:**
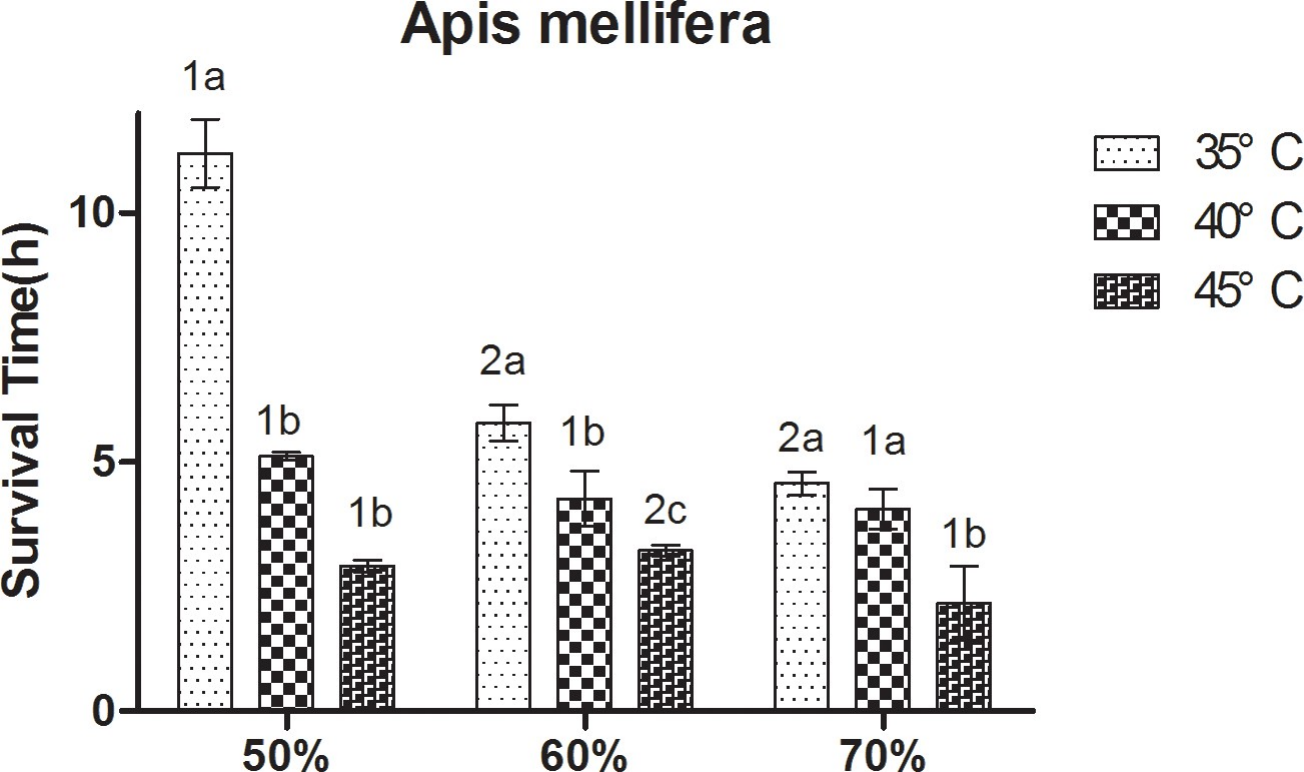
Mean ± SE survival (h) of *Apis mellifera* under different temperature and relative humidity treatments. The lowercase letters indicate significant differences between *A*. *mellifera* under different temperatures with constant humidity and the numbers indicate significant differences under different humidity levels with constant temperatures.

At constant temperature, the survival rate of bees decreased with increasing humidity. A significant (P < 0.05) and moderate positive correlation was found between the survival rate and humidity (Fig 2). At 35°C and 40°C, the survival rate of *A*. *cerana* was significantly lower at 60% RH and 70% RH than at 50% RH. At 45°C, the survival rate of *A*. *cerana* was significantly lower at 70% RH than at 50% RH and 60% RH. At 35°C, the survival rate of *A*. *mellifera* at 60% RH and 70% RH was significantly lower than at 50% RH. At 40°C, the differences between the three treatment were not significant. At 45°C, the survival rate of bees was significantly lower at 50% RH and 70% RH than at 60% RH.

Temperature had a higher effect on the survival of workers compared to humidity. The results showed that the highest reduction in worker survival rate was at temperatures of 40°C and 45°C, and RH levels of 60% and 70%. However, the reduction effect of temperature on the survival rate was higher than that of humidity.

#### Temperature tolerance

**Fig 3:**
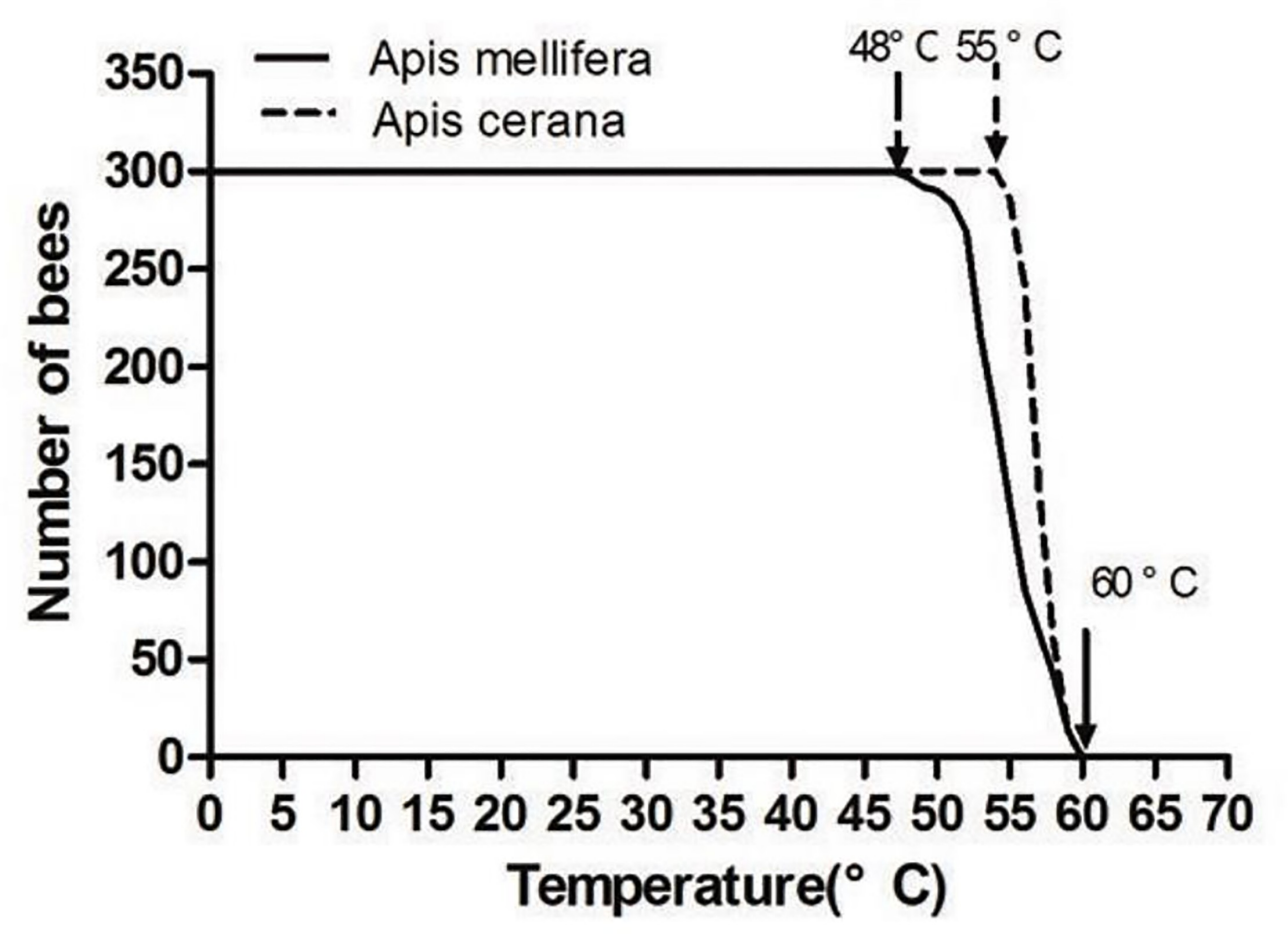
Intolerant number of *Apis cerana* and *Apis mellifera* under elevated temperature.

**Fig 4:**
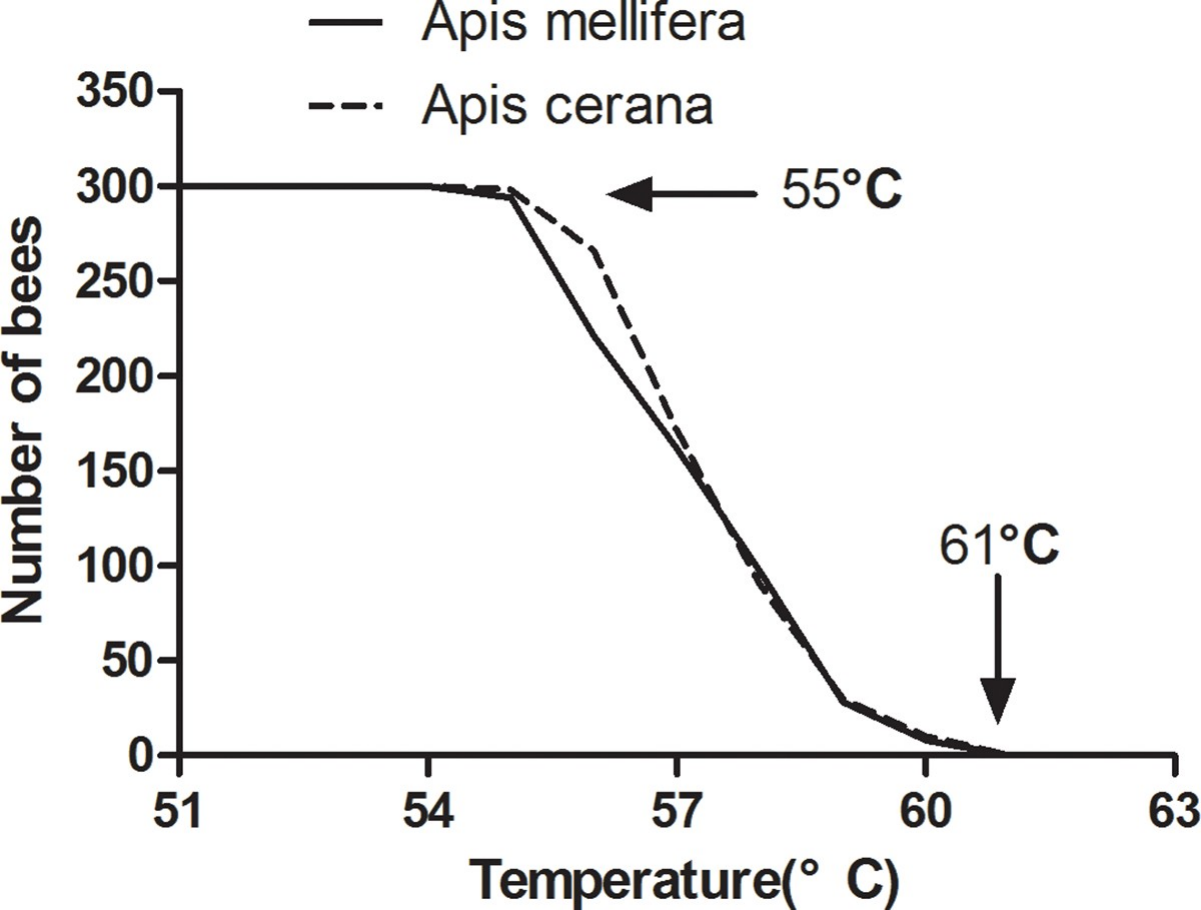
Mortality of *Apis cerana* and *Apis mellifera* under elevated temperature.

Exposure of workers bees to a temperature gradient from 30°C to 70°C showed that *A*. *mellifera* workers become intolerant at 48°C, whereas, *A*. *cerana* workers become intolerant at 55°C (Fig 3). At 61°C, both *A*. *mellifera* and *A*. *cerana* workers died (Fig 4). The intolerant temperature range for *A*. *mellifera* was from 54°C to 60°C, and that for *A*. *cerana* was from 57°C to 60°C. In general, *A*. *cerana* exhibited higher ability to tolerate temperature than that of *A*. *mellifera* under extreme conditions.

#### Loss of body water

Loss of body water in bees under different temperature and humidity conditions is shown in Table 2. At 45°C, loss of body water was highest, followed by 40°C; the least loss was at 35°C. Loss of body water of bees was lower at 60% RH and 70% RH than at 50%RH. In general, *A*. *mellifera* workers lost more body water than *A*. *cerana*, especially at 45°C,60% RH and 45°C,70% RH.

**Table 2:** Mean body water loss (mg h^–1^) of two honeybee species under different temperature and relative humidity treatments.

		*Body water loss mean (mg h*^*–1*^*) ± SE*		
RH(%)	T(°C)	*Apis cerana*	*Apis mellifera*	LSD_0.05_
50	35	1.53±0.15	2.46±0.65	0.942
	40	3.86±0.13	4.47±0.44	0.775
	45	5.23±0.21	6.63±0.51	0.665
60	35	1.47±0.37	2.19±0.15	0.115
	40	2.29±0.41	2.69±0.24	0.379
	45	4.89±0.25	5.96±0.46	0.021
70	35	1.37±0.35	2.29±0.35	0.333
	40	2.47±0.57	2.74±0.28	0.856
	45	4.99±0.46	6.09±0.64	0.023

**Fig 5:**
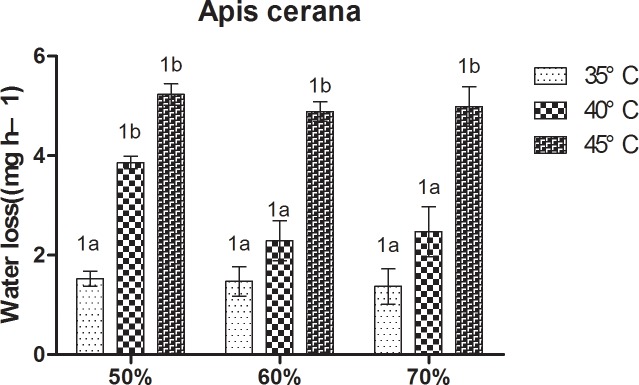
Mean ± SE body water loss (mg h^–1^) of *Apis cerana* under different temperature and relative humidity treatments. The lowercase letters indicate significant differences between *A*. *cerana* under different temperatures with constant humidity and the numbers indicate significant differences under different humidity levels with constant temperatures.

At constant humidity, loss of body water in bees increased with increasing temperature (Fig 5). At 50% RH, *A*. *cerana* showed a relatively low loss of body water at 35°C, and the highest at 45°C; loss of body water in *A*. *cerana* was significantly higher at 45°C and 40°C than at 35°C. At 60% RH and 70% RH, loss of body water in *A*. *cerana* was significantly higher at 45°C than at 35°C and 40°C. For *A*. *mellifera*, at 50% RH, it showed a relatively loss of body water at 35°C, followed by 40°C, and the highest at 45°C. Significant differences between the three treatments were observed. At 60% RH and 70% RH, loss of body water in *A*. *mellifera* was significantly higher at 45°C than 35°C and 40°C.

**Fig 6:**
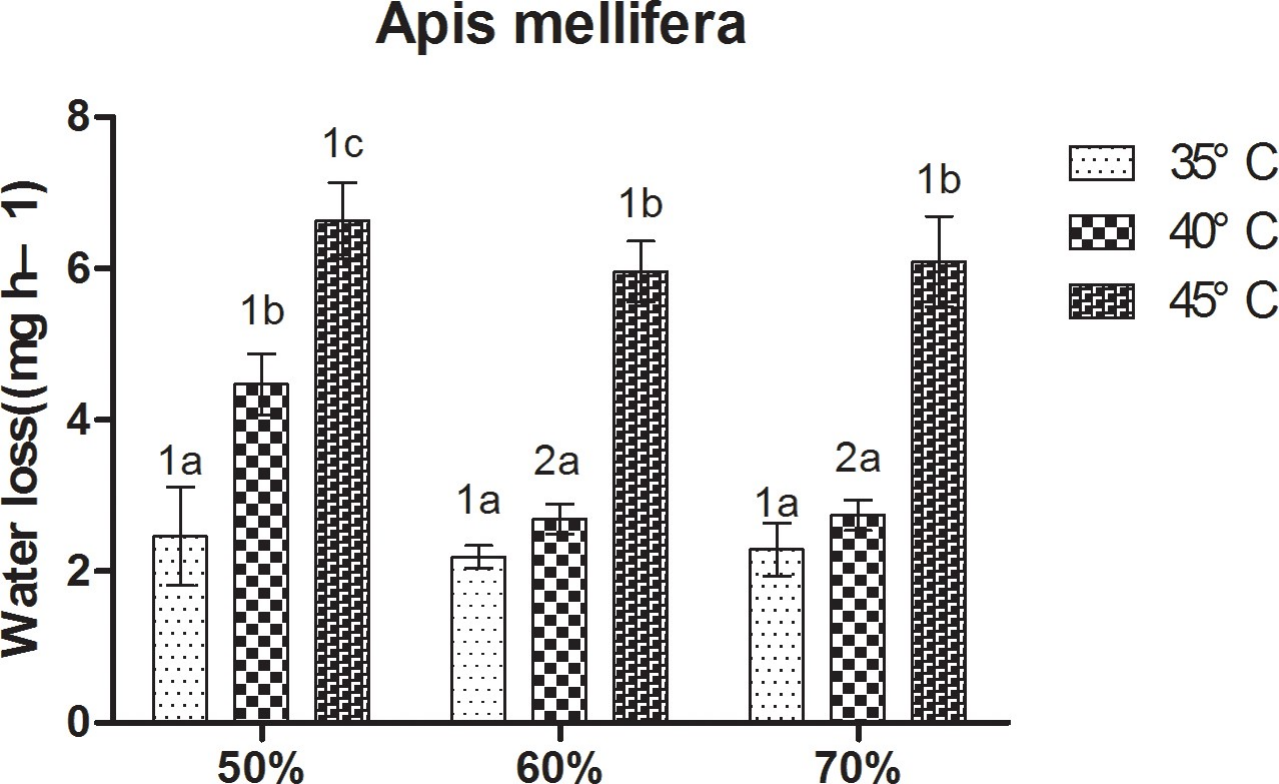
Mean ± SE body water loss (mg h^–1^) of *Apis mellifera* under different temperature and relative humidity treatments. The lowercase letters indicate significant differences between *A*. *mellifera* under different temperatures with constant humidity and the numbers indicate significant differences under different humidity levels with constant temperatures.

For *A*. *cerana*, at constant temperature, no significant differences were observed (P < 0.05) between the three treatments (Fig 6). For *A*. *mellifera*, at 40°C, the loss of body water in bees at 60% RH and 70% RH was significantly lower than at 50% RH. At 35°C and 45°C, no significant differences were observed between the three treatment.

However, considering the difference in body shape between the two species, we further compared the water loss rate of the two bees (Table 3). At 35°C, the water loss rate in *A*. *mellifera* was higher than that in *A*. *cerana*, while at 40°C and 45°C, the water loss rate in *A*. *cerana* was higher than that in *A*. *mellifera*. The water loss rate of bees at 60% RH and 70% RH was lower than that at 50% RH. The difference between the treatment groups was not significant.

**Table 3:** Mean water loss rate (%) of the two honeybee species under different temperature and relative humidity treatments.

		Body water loss mean (%) ± SE		
RH(%)	T(°C)	*Apis cerana*	*Apis mellifera*	LSD_0.05_
50	35	1.77±0.13	2.13±0.07	0.364
	40	4.48±0.31	3.87±0.04	0.136
	45	6.07±0.55	5.74±0.11	0.399
60	35	1.71±0.17	1.90±0.13	0.709
	40	2.66±0.09	2.33±0.18	0.519
	45	5.68±0.79	4.75±0.15	0.086
70	35	1.59±0.14	1.98±0.06	0.130
	40	2.87±0.17	2.37±0.21	0.059
	45	5.79±0.21	5.28±0.14	0.055

**Fig 7:**
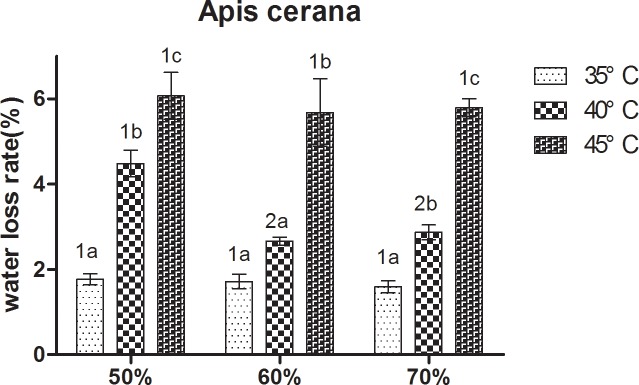
Mean ± SE water loss rate of *Apis cerana* under different temperature and relative humidity treatments (the ratio of lost water weight to total weight). The lowercase letters indicate significant differences between *A*. *cerana* under different temperatures with constant humidity and the numbers indicate significant differences under different humidity levels with constant temperatures.

At constant humidity, the water loss rate of the two bees increased with increasing temperature (Fig 7). At 50% RH and 70% RH, *A*. *cerana* showed a relatively low water loss rate at 35°C, and the highest at 45°C, Significant differences between the three treatments were observed. At 60% RH, the water loss rate in *A*. *cerana* was significantly higher at 45°C than at 35°C and 40°C. For *A*. *mellifera*, at 50% RH, it showed a relatively loss of water loss rate at 35°C, followed by 40°C, and the highest at 45°C. Significant differences between the three treatments were observed. At 60% RH and 70% RH, the water loss rate in *A*. *mellifera* was significantly higher at 45°C than 35°C and 40°C.

**Fig 8:**
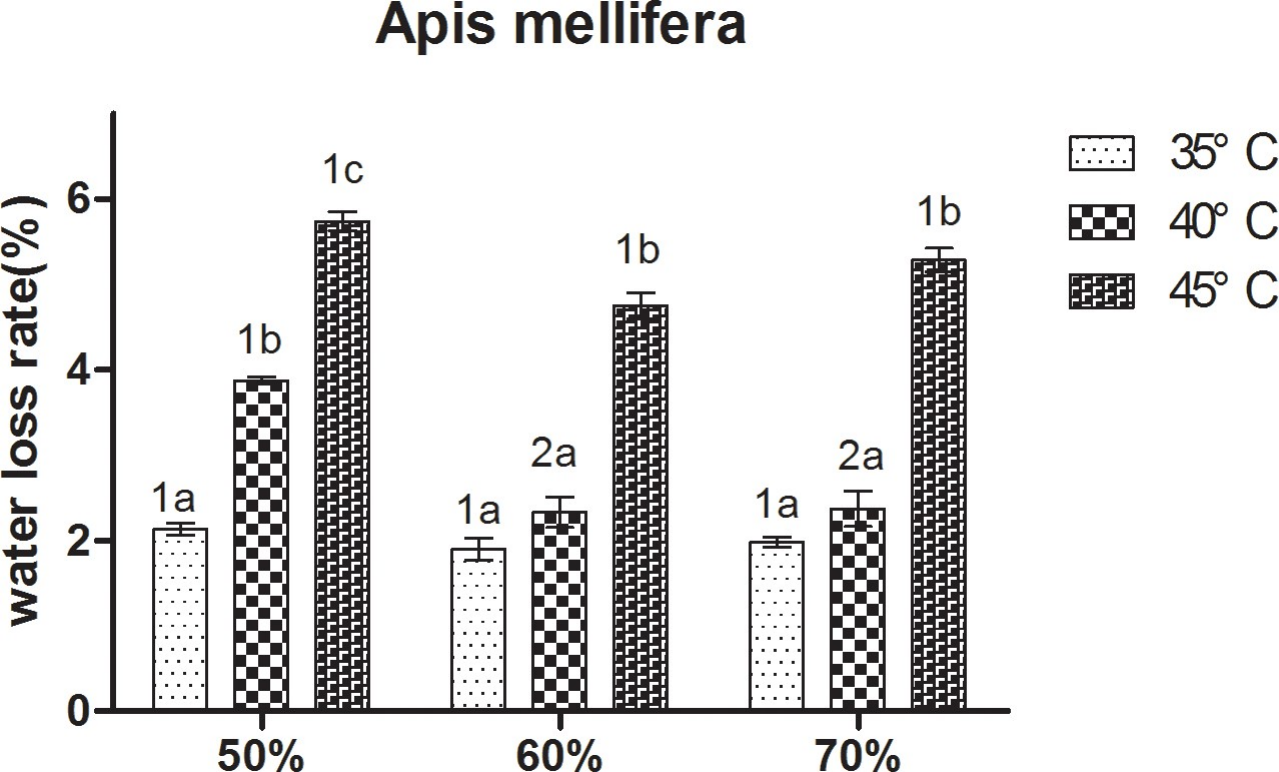
Mean ± SE water loss rate of *Apis mellifera* under different temperature and relative humidity treatments (the ratio of lost water weight to total weight). The lowercase letters indicate significant differences between *A*. *mellifera* under different temperatures with constant humidity and the numbers indicate significant differences under different humidity levels with constant temperatures.

For *A*. *cerana*, at 40°C, the water loss rate in bees at 60% RH and 70% RH was significantly lower than at 50% RH. At 35°C and 45°C, no significant differences were observed between the three treatments. The result of *A*. *mellifera* is consistent with *A*. *cerana* (Fig 8).

### Antioxidant enzyme activities

**Fig 9:**
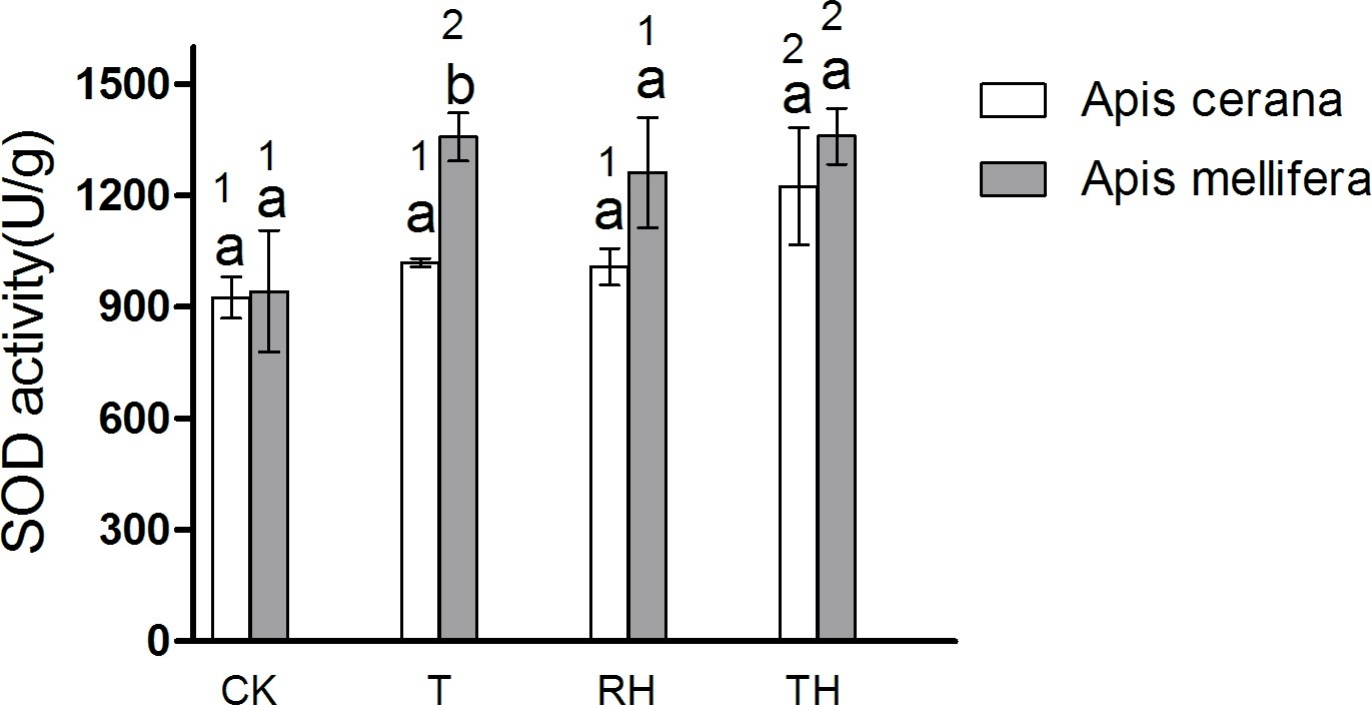
Activity of SOD treated under different temperature and humidity (CK:25°C 30% RH, RH:25°C 80% RH, T:45°C 30% RH, TH:45°C 80% RH). The lowercase letters indicate significant differences between *A*. *cerana* and *A*. *mellifera* under different temperatures with constant humidity and the numbers indicate significant differences under different humidity levels with constant temperatures.

**Fig 10:**
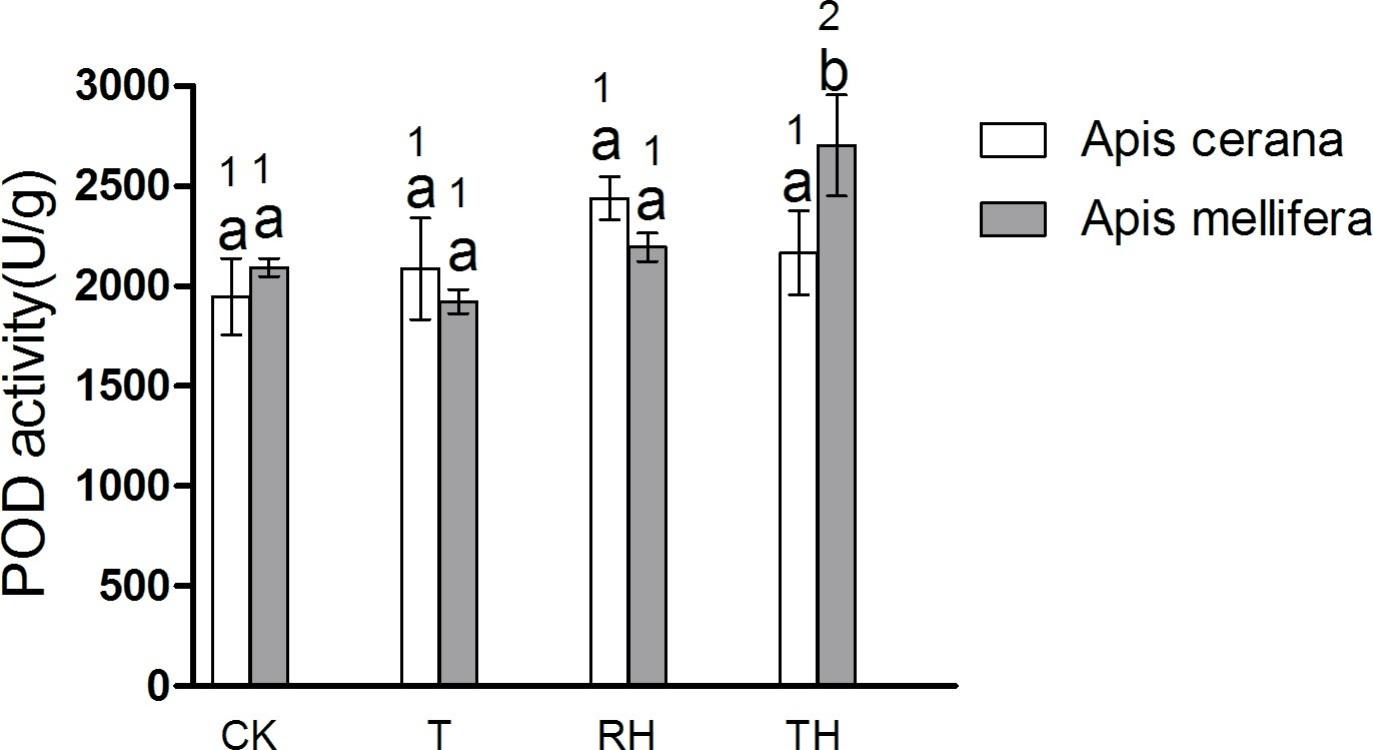
Activity of POD treated under different temperature and humidity (CK:25°C 30% RH, RH:25°C 80% RH, T:45°C 30% RH, TH:45°C 80% RH). The lowercase letters indicate significant differences between *A*. *cerana* and *A*. *mellifera* under different temperatures with constant humidity and the numbers indicate significant differences under different humidity levels with constant temperatures.

**Fig 11:**
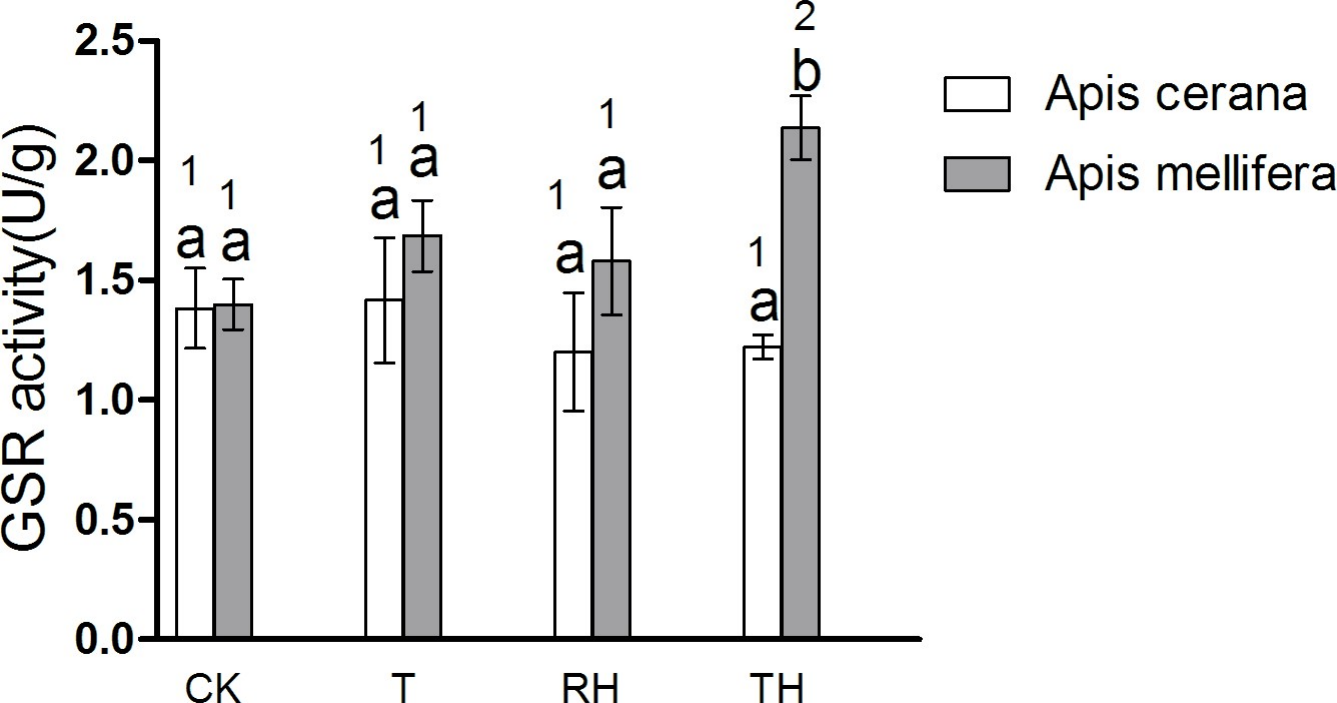
Activity of GSR treated under different temperature and humidity (CK:25°C 30% RH, RH:25°C 80% RH, T:45°C 30% RH, TH:45°C 80% RH). The lowercase letters indicate significant differences between *A*. *cerana* and *A*. *mellifera* under different temperatures with constant humidity and the numbers indicate significant differences under different humidity levels with constant temperatures.

The results showed that enzyme activity in bees under T, RH, TH treatments was higher than the control treatments. The results of *A*. *mellifera* and *A*. *cerana* were consistent. In general, the activity of antioxidant enzymes in *A*. *mellifera* was slightly higher than that in *A*. *cerana*. In the T treatment, the SOD activity of *A*. *mellifera* was significantly higher than that of *A*. *cerana* (P < 0.05) (Fig 9). In the TH treatment, the activity of POD and GSR of *A*. *mellifera* was significantly higher than that of *A*. *cerana* (P < 0.05)(Figs 10 and 11).

**Fig 12:**
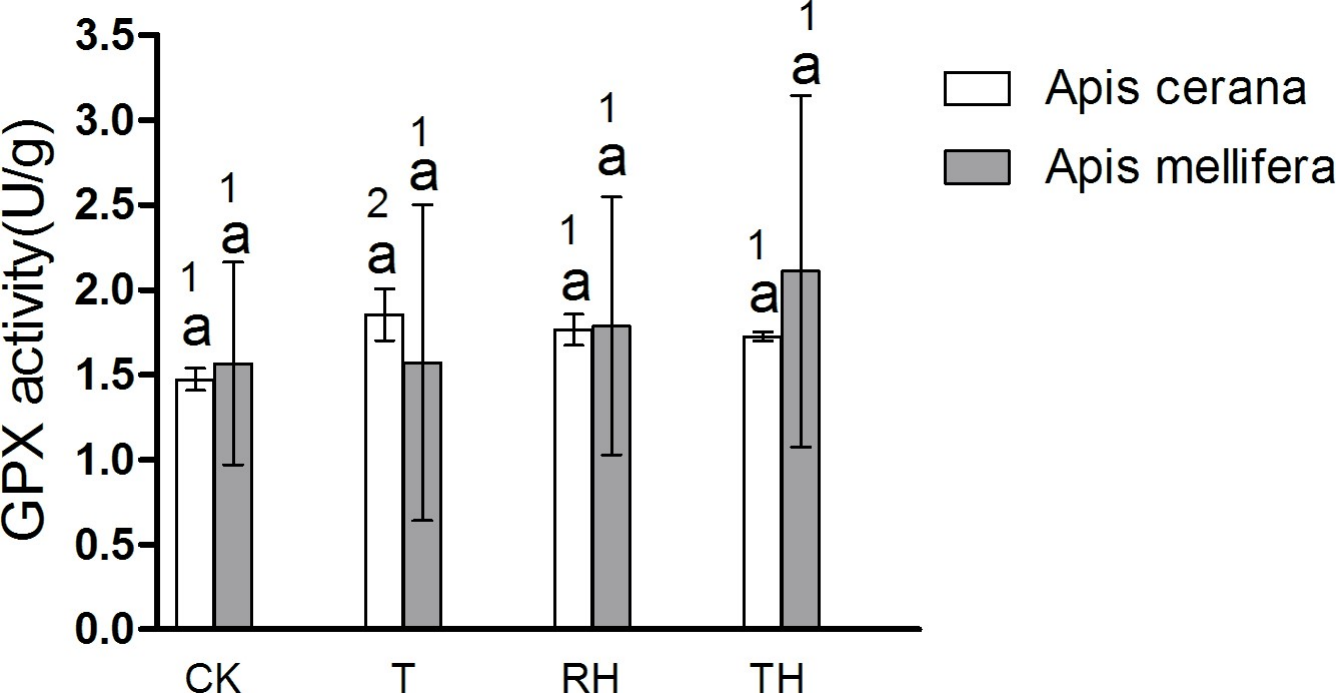
Activity of GPX treated under different temperature and humidity (CK:25°C 30% RH, RH:25°C 80% RH, T:45°C 30% RH, TH:45°C 80% RH). The lowercase letters indicate significant differences between *A*. *cerana* and *A*. *mellifera* under different temperatures with constant humidity and the numbers indicate significant differences under different humidity levels with constant temperatures.

**Fig 13:**
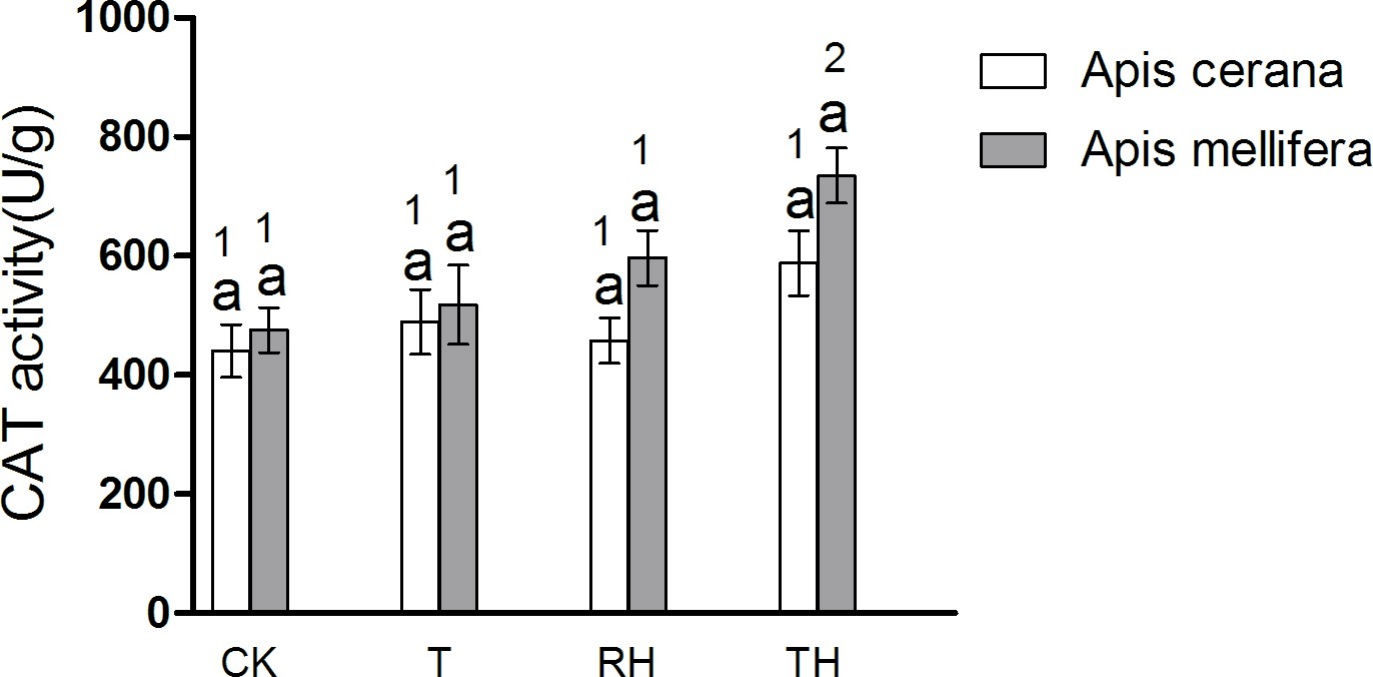
Activity of CAT treated under different temperature and humidity (CK:25°C 30% RH, RH:25°C 80% RH, T:45°C 30% RH, TH:45°C 80% RH). The lowercase letters indicate significant differences between *A*. *cerana* and *A*. *mellifera* under different temperatures with constant humidity and the numbers indicate significant differences under different humidity levels with constant temperatures.

In *A*. *cerana*, the SOD activity of TH treatment was significantly higher than the other treatments (P < 0.05)(Fig 9); the GPX activity of T treatment was significantly higher than the other treatments (P < 0.05)(Fig 12); and for CAT, POD, and GSR, the difference between treatment groups was not significant. In *A*. *mellifera*, The SOD activity of T, TH treatment was significantly higher than the other treatments (P < 0.05)(Fig 9); the POD, GSR, CAT activity of TH treatment was significantly higher than the other treatments (P < 0.05)(Figs 10, 11 and 13); and for GPX, the difference between treatment groups was not significant.

### Detoxification enzymes activity

**Fig 14:**
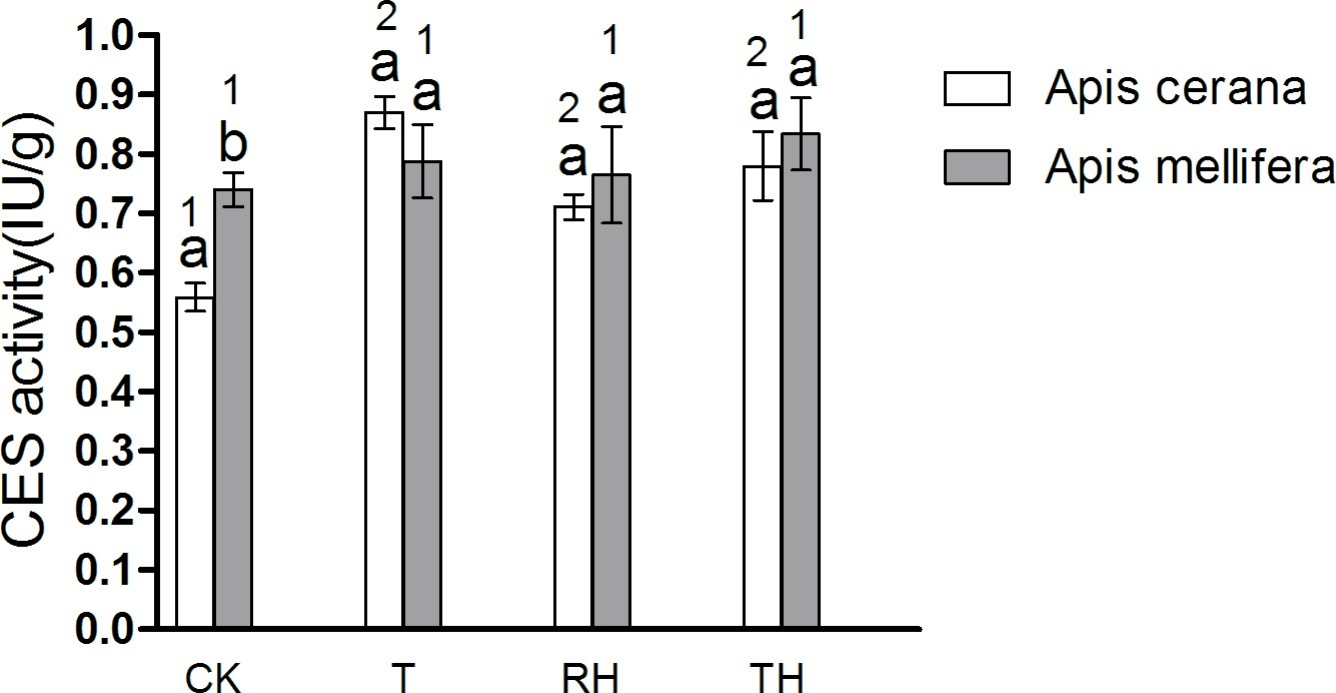
Activity of CES treated under different temperature and humidity (CK:25°C 30% RH, RH:25°C 80% RH, T:45°C 30% RH, TH:45°C 80% RH). The lowercase letters indicate significant differences between *A*. *cerana* and *A*. *mellifera* under different temperatures with constant humidity and the numbers indicate significant differences under different humidity levels with constant temperatures.

We measured the activity of five detoxification enzymes. The results were consistent with those of antioxidant enzyme activities. Enzyme activity in honeybees subjected to different treatments was higher than that of the controls. The results of *A*. *mellifera* and *A*. *cerana* were consistent. Overall, the detoxification enzyme activity of *A*. *mellifera* was higher than that of *A*. *cerana*. In the CK treatment, the CES activity of *A*. *mellifera* was significantly higher than that of *A*. *cerana* (P < 0.05) (Fig 14).

**Fig 15:**
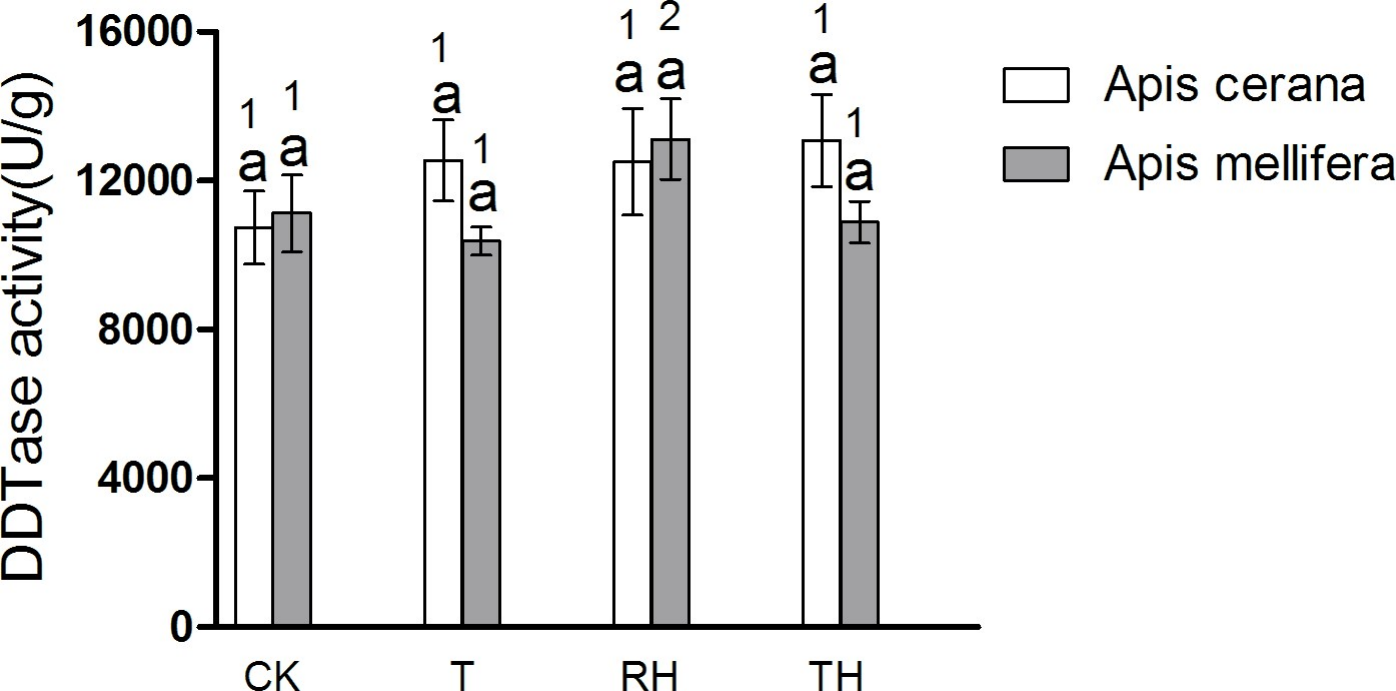
Activity of DDTase treated under different temperature and humidity (CK:25°C 30% RH, RH:25°C 80% RH, T:45°C 30% RH, TH:45°C 80% RH). The lowercase letters indicate significant differences between *A*. *cerana* and *A*. *mellifera* under different temperatures with constant humidity and the numbers indicate significant differences under different humidity levels with constant temperatures.

In *A*. *cerana*, the CES activity of CK treatment was significantly lower than the other treatments (P < 0.05) (Fig 14); and for DDTase, GST, CYP450, and AChE, the difference between treatment groups was not significant. In *A*. *mellifera*. The DDTase activity of RH treatment was significantly higher than the other treatments (P < 0.05) (Fig 15); and for GST, CES, CYP450, and AChE, the difference between treatment groups was not significant.

## Discussion

### Epiphysiology

In the long-term evolution process, insects have evolved a series of behavioral responses and physiological mechanisms to adapt to extreme environmental temperatures [25]. Survival, upper lethal limit, and water loss are the main physiological characteristics of insects in extreme temperatures. We studied these characteristics of two closely related bee species (*A*. *mellifera* and *A*. *cerana*) living in different climate regions. These two bees can be used to study other species with long-term adaptation to specific environments in order to determine their particular physiological adaptability [26]. The present study showed that there may be differences between species due to adaptation to different climates. Our findings on the physiological characteristics of bees will provide a methodological reference for future studies on these characteristics of bees.

In our study, high temperature environment exhibited a negative effect on the survival of honeybees. This result is consistent with those of previous studies. Mardan and Kevan [27] found that *Apis dorsata* can survive for 5 days and 48 hours at 38° C and 45° C, respectively. Free and Spencer-Booth [28] indicated that the survival of bees at high temperatures depends on the duration of exposure and the relative humidity. At higher temperatures, they only survive at lower relative humidity for a short period of time because the temperature decreases by evaporation of water evaporation [29]. Abou-Shaara [30] investigated the tolerance of Yemen bees (adapted to harsh conditions) and Carniolan bees to high temperature and low humidity conditions in the desert. Their results showed that compared to relative humidity, temperature has a greater impact on workers' survival. In most cases, the response of the two bees in the treatments is somewhat similar, and the Yemen bees are more tolerant than the Carniolan bees only under extreme conditions of high or low humidity.

The present study showed that the average survival rate of worker bees was highest at 35°C, and *A*. *mellifera* showed higher survival than that of *A*. *cerana* at 40°C and 45°C. These results indicate that *A*. *mellifera* seems to be more suitable for high temperature and high humidity than *A*. *cerana*. According to Joshi, the effect of humidity on the bees was negligible [13]. However, our results show that RH has an effect on the survival rate of bees, although this effect is less than temperature. We speculate that the effect of high humidity on bees might be in association with high temperature. At relatively high temperatures, high humidity can inhibit water loss in bees, and therefore, body temperature of bees is not reduce effectively, consequently damaging the body.

The results showed that *A*. *cerana* seem to be more adaptable to extreme heat than *A*. *mellifera*. Abou-Shaara [30] showed that Yemen bees are more tolerant than Carniolan bees under extreme conditions. Kovac and Käfer [31] found that Ligustica bees had higher tolerance and survival at high temperatures (with lethal temperatures (LT 50, 8 h) of Carniolan bees being 50.3°C and Ligustica bees being 51.7°C. Furthermore, Mardan and Kevan [27] reported that temperatures between 26°C and 36°C did not affect the survival rate of bees. Continued warming can affect the survival rate of worker bees, as observed in the present study. This indicates the poor ability of bees to withstand high temperatures for a long period.

In this study, *A*. *mellifera* was mostly intolerant at 54–60°C, and *A*. *cerana* at 57–60°C. In extreme environments, tolerance of *A*. *cerana* was stronger than that of *A*. *mellifera*. Atmowidjojo [24] found that worker bees were the most tolerant at temperatures of 50.7°C. This finding is not consistent with the result of the present study. These differences could be attributed to different experimental conditions and bee species. The water loss rate experiment seems to explain the results of our experiments. We found that the water loss rate of the *A*. *cerana* was higher than that of the *A*. *mellifera* under extreme high temperature, indicating that the cooling efficiency of the *A*. *cerana* was better and the tolerance was stronger under extreme high temperature.

As an adaptation to avoid high temperatures, insects first reduce the body and body surface temperature by increasing the evaporation of water from the body to avoid high temperature damage. Evaporative cooling with water droplets is a common behavior of bees to reduce body temperature at high ambient temperatures [31]. Heinrich [29] found that when bees were at ambient temperatures of 46°C, the temperature of the head was approximately 43°C, indicating that bees lower their body temperature at high temperatures, and that evaporation aids in decreasing the temperature.

On the basis of our results, we believe that *A*. *mellifera* seem to be better adapted to high temperatures and humidity than *A*. *cerana* as *A*. *mellifera* presented more body water loss than *A*. *cerana* under different temperature and humidity treatments. This is consistent with the findings of Al-Qarni [32], who found that the average weight loss of *A*. *mellifera* was higher than that of *A*. *cerana* exposed to a higher temperature for 2 h. However, considering the difference in body shape between the two bees, we further studied the water loss rate of the two bees, and found that under the extreme high temperatures of 40°C and 45°C, the water loss rate of *A*. *cerana* was higher than that of *A*. *mellifera*. This can be explained by the observation that extreme high temperature tolerance of the *A*. *cerana* in the tolerance experiment was higher than that of the *A*. *mellifera*. In addition, in the present study, the water loss rate increased with the increase in temperature. This is consistent with the finding of Roberts and Harrison [33], who observed an increase in water loss after exposure of bees to 33°C.

### Enzyme activities

Antioxidant enzymes are a type of defensive enzymes in bees, which can effectively remove the superoxide free radicals produced by the body during the metabolism process, thus eliminating the toxic effects on cells. Antioxidant enzymes play an important role in scavenging free radicals, preventing oxygen free radicals from damaging the composition, structure and function of cells, and protecting cells from oxidative damage. Therefore, they play an important role in maintaining the balance of active oxygen metabolism in bees [34].

Investigating the antioxidant response of insects crucial for understanding their physiological and biochemical aspects in response to temperature stress [19]. To determine the oxidative stress caused by changes in temperature and humidity on bees and the antioxidant capacity of bees, we determined the activity of antioxidant enzymes in bees. Peroxidase (CAT), glutathione-s-transferase (GSTs), peroxidase (POD), and superoxide dismutase (SOD) are several important antioxidant enzymes that act synergistically to resist the oxidative stress caused by high concentrations of reactive oxygen species (ROS) in cells. The results showed that temperature stress increased the activity of antioxidant enzymes in bees indicating that heat stress caused severe oxidative stress on bees. Meanwhile, this indicates that the bee’s antioxidant system are highly sensitive to high temperature stress and can respond in time. The reaction, in which SOD, CAT, and POD activity are significantly increased, plays an active role in resisting oxidative stress caused by heat stress. This is similar to the finding of Yang and Huang [20], who found that in citrus red mite under high temperature stress, the body’s antioxidant enzyme activity was significantly improved. Interestingly, higher humidity also led to an increase in bee antioxidant enzyme activity, although the difference was not significant compared to the control. We believe that higher humidity will prevents the bees from losing water quickly and lowering body temperature, thereby causing a certain degree of oxidative stress on the bees.

With respect to endogenous and exogenous toxic substances, the detoxification enzyme system can effectively remove them and protect the body from damage. The antioxidant enzyme system and detoxification enzyme system are an important stress-resistance mechanisms for biological response to various environmental stresses; they play an important role in the process of biological adaptation to the environment [35].

Clofenotane dehydrochlorinase (DDTase), glutathione S-transferase (GST), carboxylesterase (CES), cytochromeP450 (CYP450P), and acetyl cholinesterase (AChE) are several important detoxifying enzymes. They have been used as a biomarkers to assess the production of toxic substances in an organism under oxidative stress. In order to understand the response of detoxification enzymes to honeybees under high temperature and high humidity stress, we determined the activity of several detoxifying enzymes in bees. The results showed that high temperature and high humidity can improve the detoxification enzyme activity of bees, which indicates that the high temperature or high humidity can cause oxidative stress and metabolic poisons of bees. This suggests that the honeybee's detoxification enzyme system is responsive to oxidative stress caused by heat stress. A variety of detoxifying enzymes in bees can synergistically eliminate metabolic poisons damage to bees.

Comprehensive analysis showed that heat stress caused an imbalance of redox metabolism in bees, and caused oxidative stress. This indicates that heat stress has caused damage to bees and may be the most important factor in generating oxidative stress. Under this oxidative stress, antioxidant enzymes exhibit positive resistance to protection. This indicates that they may be an important mechanism of anti-oxidative stress of bees, and also reflect certain physiological adaptability. *A*. *mellifera* appeared to have more powerful antioxidant capacity than *A*. *cerana*. This is consistent with previous experimental results that *A*. *mellifera* survived at high temperatures for a longer period of time.

High temperature and high humidity have a negative effect on workers’ survival. It is reasonable to believe that *A*. *mellifera* are more adaptable under greenhouse conditions, high temperatures, and high humidity. We believe that the effect of high humidity on bees is the result of collaboration with high temperatures. The effect of humidity as a single factor is not yet clear. In addition, whether the effect of high temperature and high humidity on young bees is consistent with adult bees requires further investigation. The findings of this study provide a theoretical basis for investigating the stress resistance mechanism of honeybees under high temperature and humidity conditions. The study also highlights the importance of regulating temperature and humidity in agricultural production facilities and the protection of pollination bee colonies.
